# Computed tomography-derived sarcopenia, enteral nutritional support, and febrile neutropenia burden in children with acute lymphoblastic leukemia: a retrospective cohort study

**DOI:** 10.3389/fped.2026.1752343

**Published:** 2026-05-18

**Authors:** Şule Çalışkan Kamış, Burak Kamış, Metin Çil, Nihal Boz, Ayşe Selcan Koç, Begül Yağcı

**Affiliations:** 1 Department of Pediatric Hematology and Oncology, Adana Faculty of Medicine, Adana City Training and Research Hospital, University of Health Sciences, Adana, Türkiye; 2 Department of Child and Adolescent Psychiatry, Adana Faculty of Medicine, Adana City Training and Research Hospital, University of Health Sciences, Adana, Türkiye; 3 Department of Radiology, Adana Faculty of Medicine, Adana City Training and Research Hospital, University of Health Sciences, Adana, Türkiye

**Keywords:** acute lymphoblastic leukemia, enteral nutritional support, febrile neutropenia, sarcopenia, total psoas muscle area

## Abstract

**Objective:**

This study aimed to evaluate computed tomography-derived sarcopenia and enteral nutritional support in relation to febrile neutropenia burden, hospital stay, and mortality in children receiving treatment for acute lymphoblastic leukemia.

**Materials and methods:**

This retrospective single-center cohort included 100 children aged 3–17 years with acute lymphoblastic leukemia treated between April 2020 and March 2024. Clinical data on febrile neutropenia episodes, neutropenia duration, severe neutropenia, hospital stay, enteral nutritional support, and febrile neutropenia-related mortality were collected from hospital records. Sarcopenia analysis was restricted to 50 patients with clinically indicated computed tomography images suitable for total psoas muscle area measurement. Total psoas muscle area was measured at the L4–L5 level. Sarcopenia was defined as a total psoas muscle area z-score < −2 according to age- and sex-specific reference values.

**Results:**

The median number of febrile neutropenia episodes was 6.5, ranging from 1 to 19. Febrile neutropenia-related mortality was observed in 17 patients. Forty-five patients received enteral nutritional support. Enteral nutritional support was associated with higher febrile neutropenia burden in unadjusted analysis; however, this was not interpreted as causal because enteral feeding may reflect underlying clinical severity. Among the 50 patients with total psoas muscle area data, 40 patients, corresponding to 80% of the imaged subgroup, were sarcopenic. Sarcopenia was not significantly associated with febrile neutropenia frequency, severe neutropenia, mortality, or hospital stay. In exploratory adjusted analysis, enteral nutritional support showed a borderline but non-significant association with febrile neutropenia frequency (IRR: 1.16, 95% CI: 1.00–1.36, *p* = 0.052).

**Conclusion:**

Computed tomography-derived sarcopenia was common among imaged children with acute lymphoblastic leukemia but was not independently associated with febrile neutropenia burden or hospital stay. Enteral nutritional support should be interpreted as a marker of clinical vulnerability rather than a causal determinant of febrile neutropenia.

## Introduction

Sarcopenia is defined by the loss of muscle mass and function in the body. Although it is well established in adults, sarcopenia remains an evolving concept in pediatric populations because muscle mass is strongly influenced by age, sex, growth, pubertal development, and underlying disease. Inflammatory conditions, such as acute leukemia, can exacerbate muscle damage through chronic inflammation, metabolic stress, reduced physical activity, and treatment-related toxicity, thereby increasing the risk of sarcopenia. Febrile neutropenia, a common complication during cancer treatments such as chemotherapy, may further contribute to functional decline by increasing hospitalization, limiting physical activity, and promoting catabolic stress.

Sarcopenia often arises as a secondary consequence of systemic inflammatory diseases such as cancer and is a frequent finding in children with cancer, adversely affecting their prognosis. With the widespread use of radiological imaging in clinical settings, the rapid assessment of body composition has become feasible, enabling early detection of lean mass loss in pediatric cancer patients (1–3).

Pediatric malnutrition leads to adverse effects on growth, neurocognitive development, and body functions due to nutritional imbalance. In children with cancer, malnutrition may result from increased energy requirements, reduced oral intake, mucositis, vomiting, diarrhea, gastrointestinal toxicity, systemic inflammation, and treatment-related catabolism. While sarcopenia is defined as loss of muscle mass and strength in adults, it is a newer concept in children. The malnutrition-sarcopenia syndrome refers to the co-occurrence of these two conditions.

However, body weight alone does not adequately define nutritional status in pediatric oncology patients because it does not distinguish between lean mass, fat mass, fluid retention, and treatment-related changes. In children, sarcopenia is associated with growth delays and neurodevelopmental issues. Diagnosis of sarcopenia typically focuses on muscle mass and functionality, which are directly influenced by the inflammatory effects of chronic diseases (4–6).

Anthropometric methods used to assess sarcopenia measure muscle mass indirectly and may provide erroneous results, especially in children at risk of fluid overload. Computed tomography and magnetic resonance imaging are considered reliable modalities for directly measuring skeletal muscle mass. In pediatric oncology, total psoas muscle area derived from routine computed tomography images has been increasingly used as an imaging-based marker of low muscle mass. When evaluating pediatric sarcopenia, it is important to consider growth and developmental factors as well (7, 8).

In children with cancer, malnutrition, which involves increased energy needs and reduced nutrient intake, can lead to sarcopenia, potentially negatively affecting the patient's prognosis (9, 10). However, standardized nutritional assessment requires anthropometric z-scores, dietary evaluation, weight change during treatment, or validated malnutrition screening tools. In the present study, such comprehensive nutritional scoring was not available for all patients. Therefore, enteral nutritional support was analyzed as a supportive-care variable reflecting the need for nutritional intervention during therapy, rather than as a direct marker of malnutrition.

In this study, we aimed to evaluate the prevalence of computed tomography-derived sarcopenia and to examine its association with febrile neutropenia episodes, hospital stay, and mortality in children undergoing treatment for acute lymphoblastic leukemia.

We also explored the relationship between enteral nutritional support and febrile neutropenia burden, while avoiding causal interpretation because enteral feeding may reflect underlying clinical severity.

## Materials and methods

This retrospective, single-center cohort study included children aged 3–17 years who were treated for acute lymphoblastic leukemia at Adana City Training and Research Hospital between April 1, 2020, and March 27, 2024. Patients younger than 18 years were screened; after exclusion of patients with incomplete records, the final cohort consisted of 100 patients aged 3–17 years. Measurements of paravertebral muscle and psoas muscle thickness and width were taken from routine computed tomography images obtained for clinical indications during treatment. Computed tomography scans were not performed for research purposes.

The number of febrile neutropenia episodes and the use of enteral nutritional support were reviewed from the hospital system. This study evaluated computed tomography-derived sarcopenia and enteral nutritional support in relation to febrile neutropenia episodes, neutropenia-related outcomes, hospital stay, and mortality in children treated for acute lymphoblastic leukemia.

The patients' date of birth, age, sex, diagnosis, body weight, paravertebral muscle and psoas muscle thickness and width, use of enteral nutritional support, number of febrile neutropenia episodes, duration of neutropenia, duration of severe neutropenia, hospital stay, and febrile neutropenia-related mortality were recorded. The parameters used in the study were obtained from routine clinical examination and treatment records. Patients with incomplete data in hospital records were excluded from the study. Patients aged 18 years or older were not included.

The overall cohort included 100 patients with complete clinical data. Sarcopenia-specific analyses were restricted to the 50 patients who had clinically indicated computed tomography images suitable for total psoas muscle area measurement. Patients without suitable computed tomography images were not included in sarcopenia-specific analyses. Therefore, sarcopenia findings represent the computed tomography-imaged subgroup and should not be generalized to the entire cohort.

Enteral nutritional support was defined as the use of oral or enteral nutritional formulas prescribed during treatment because of inadequate oral intake, nutritional risk, gastrointestinal toxicity, mucositis, or clinician-determined need for nutritional support. Enteral nutritional support was not considered a direct marker of malnutrition; rather, it was analyzed as a supportive-care variable reflecting the need for nutritional intervention during therapy.

Febrile neutropenia was defined as fever accompanied by neutropenia, according to institutional clinical practice. Fever was defined as a single axillary temperature of ≥38.0 °C or a sustained temperature of ≥37.5 °C for at least one hour. Neutropenia was defined as an absolute neutrophil count below 500/mm^3^ or expected to decrease below 500/mm^3^ within 48 h. Severe neutropenia was defined as an absolute neutrophil count below 500/mm^3^. The number of febrile neutropenia episodes was determined from hospital records. Neutropenia duration was calculated using serial complete blood count results obtained during hospitalization and follow-up visits.

### Psoas muscle area measurement

**Figure 1:**
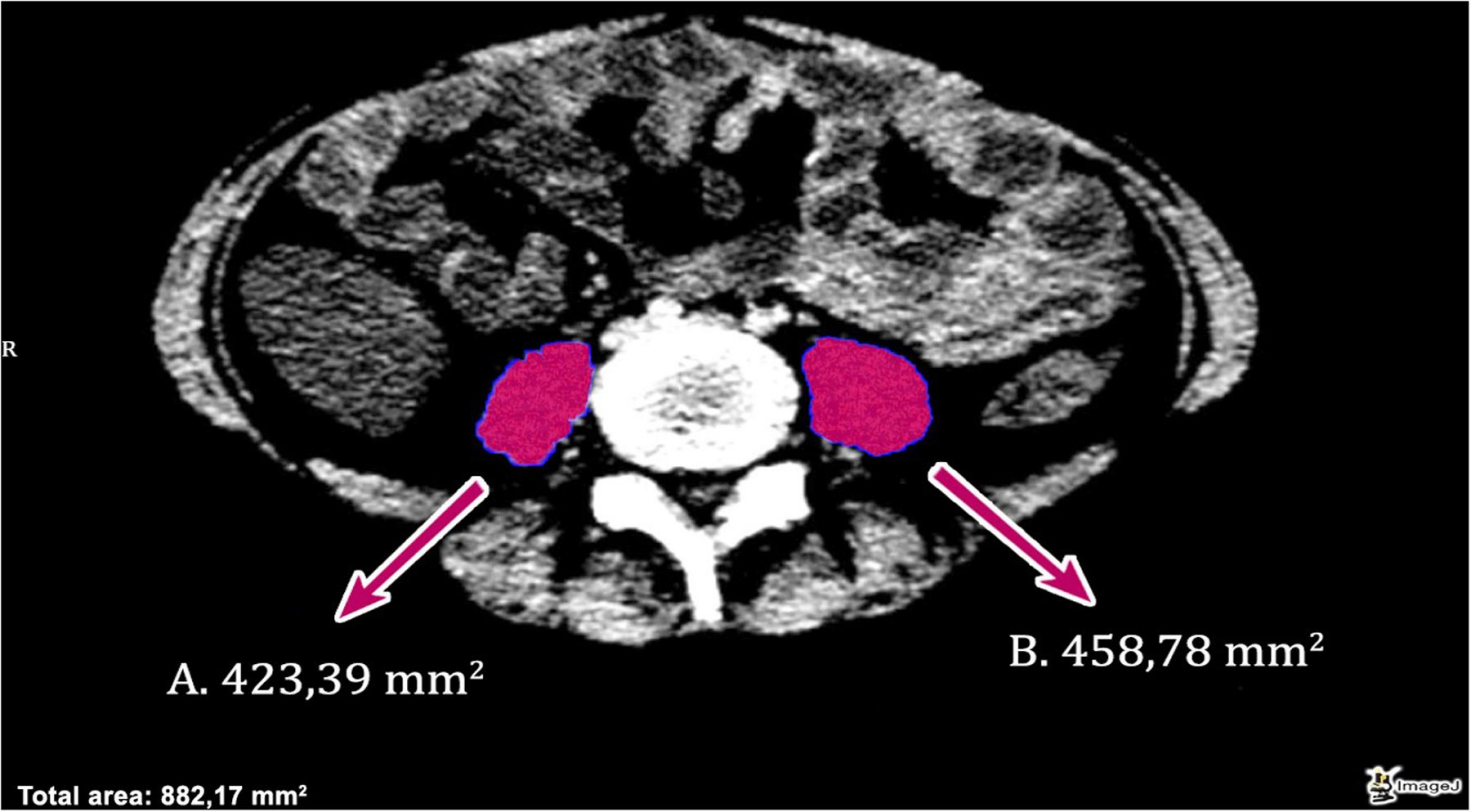
Representative axial computed tomography image from one patient included in the study, illustrating bilateral total psoas muscle area measurement at the L4–L5 intervertebral disc level. The left psoas muscle area **(A)** was measured as 423.39 mm^2^, and the right psoas muscle area **(B)** was measured as 458.78 mm^2^. The total psoas muscle area at this level was 882.17 mm^2^. The highlighted regions show the manually delineated psoas muscle contours used for sarcopenia assessment.

Total psoas muscle area (tPMA) was measured at the L4–L5 intervertebral disc level by summing the cross-sectional areas of the right and left psoas muscles, as shown in Figure 1. The muscle boundaries were manually delineated on axial computed tomography images, and the areas were expressed in square millimeters.

Age- and sex-specific percentile curves reported by Lurz et al. in 2020 were used to evaluate total psoas muscle area. Z-scores were calculated using the online calculator developed by Lurz et al. (11). Based on the calculated z-score results, patients were classified as sarcopenic or non-sarcopenic. Individual z-score values were not entered into the statistical dataset as a separate continuous variable; however, the derived categorical sarcopenia variable was recorded and used for analysis. Sarcopenia was defined as a total psoas muscle area z-score < **−2**.

**Figure 2:**
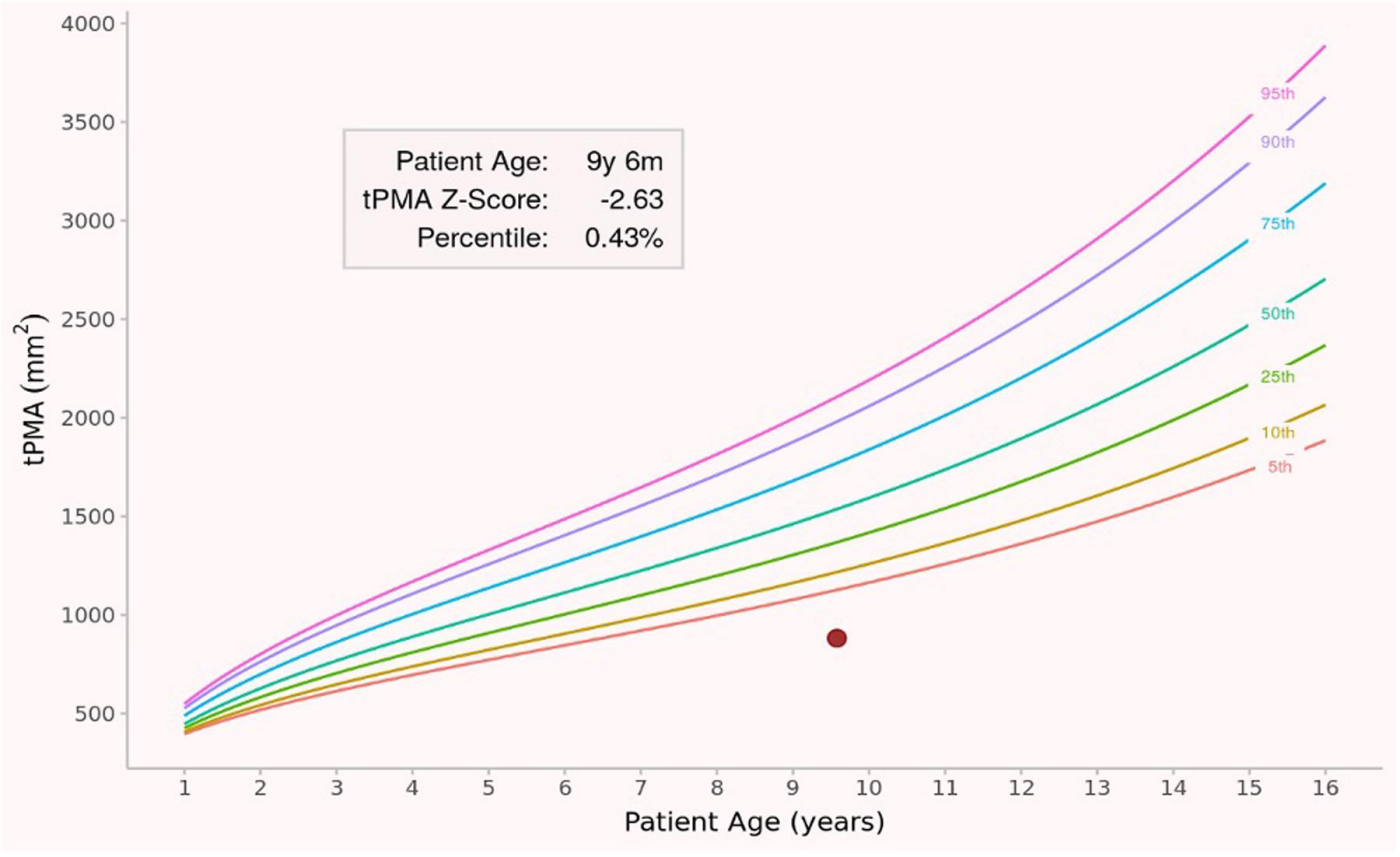
Representative percentile plot from one patient included in the study, demonstrating total psoas muscle area in relation to age- and sex-specific reference curves reported by Lurz et al. The red dot indicates the individual patient's total psoas muscle area measurement at the age of 9 years and 6 months. The calculated total psoas muscle area z-score was −2.63, corresponding to the 0.43rd percentile, consistent with sarcopenia according to the predefined cutoff of z-score < −2.

Individual tPMA measurements were then plotted on the reference percentile curves, as illustrated in Figure 2, to determine each patient's percentile ranking and deviation from the population norm. In pediatric patients, the psoas muscle at the L4–L5 level typically shows a rounder cross-sectional shape, which improves contour delineation and interrater reliability. Since the L4–L5 level is also the reference point for visceral adipose tissue assessment, measurements at this level were considered reliable indicators of skeletal muscle mass in the present study.

This retrospective study was approved by the Adana City Training and Research Hospital Clinical Research Ethics Committee on March 28, 2024, with decision number 3253. The study was conducted in accordance with the principles of the Declaration of Helsinki.

### Statistical analysis

The data were analyzed using SPSS (Statistical Package for the Social Sciences) version 26.0. Categorical variables were presented as numbers and percentages, while numerical variables were presented as mean and standard deviation or median and range, depending on data distribution. The normality of numerical variables was assessed using the Kolmogorov–Smirnov test.

The Mann–Whitney U test was used for the comparison of non-normally distributed numerical variables between two independent groups, while Student's *t*-test was used for normally distributed variables. The chi-square test or Fisher's exact test was used, as appropriate, for comparisons of categorical variables between groups. The Kruskal–Wallis test was used to compare numerical variables among three or more independent groups when the data were not normally distributed. Correlation analysis was performed to assess relationships between variables. Pearson correlation analysis was used for normally distributed variables, whereas Spearman correlation analysis was used for non-normally distributed variables.

The overall cohort of 100 patients was used for descriptive clinical analyses. Analyses involving total psoas muscle area and sarcopenia were restricted to the 50 patients with available computed tomography-derived total psoas muscle area measurements. Patients without suitable computed tomography images were not included in sarcopenia-specific analyses.

Exploratory multivariable analyses adjusted for age, sex, and body weight were performed to evaluate whether enteral nutritional support or sarcopenia was independently associated with febrile neutropenia episode frequency and hospital stay. Poisson regression was used for febrile neutropenia episode counts, and linear regression was used for hospital stay duration. For regression analyses, effect estimates were reported as incidence rate ratios for Poisson regression and beta coefficients for linear regression, with 95% confidence intervals. Because sarcopenia data were available only for 50 patients and the non-sarcopenic subgroup was small, these analyses were interpreted cautiously.

A *p* value of ≤0.05 was considered statistically significant.

## Results

The study included 100 patients aged 3–17 years who were treated for acute lymphoblastic leukemia at Adana City Training and Research Hospital between April 1, 2020, and March 27, 2024. The patients consisted of 42 girls (42%) and 58 boys (58%). The median age of the patients at the time of diagnosis was 8 years, ranging from 3 to 17 years. The median body weight of the patients was 20 kg, ranging from 9 to 69 kg.

The median number of febrile neutropenia episodes was 6.5, ranging from 1 to 19 episodes. The mean duration of neutropenia was 11 days, ranging from 2 to 23 days, and the mean duration of severe neutropenia was 9 days, ranging from 1 to 18 days. Forty-five patients (45%) received enteral nutritional support, whereas 55 patients (55%) did not. Febrile neutropenia-related mortality was observed in 17 patients (17%).

Computed tomography images suitable for paravertebral and psoas muscle measurements were available in 50 of the 100 patients. Therefore, sarcopenia-specific analyses were restricted to these 50 patients. Patients without suitable computed tomography images were not included in sarcopenia-specific comparisons.

Among the 50 patients with available computed tomography images, the median anteroposterior diameter of the right paravertebral muscle was 21 mm, ranging from 10 to 37 mm, and the median mediolateral diameter was 45 mm, ranging from 31 to 62 mm. For the left paravertebral muscle, the median anteroposterior diameter was 21 mm, ranging from 10 to 36 mm, and the median mediolateral diameter was 46.5 mm, ranging from 31 to 62 mm. The median anteroposterior diameter of the right psoas muscle was 29.5 mm, ranging from 18 to 42 mm, and the median mediolateral diameter was 9.2 mm, ranging from 3.5 to 20 mm. For the left psoas muscle, the median anteroposterior diameter was 30 mm, ranging from 18 to 42 mm, and the median mediolateral diameter was 9 mm, ranging from 3.5 to 19 mm.

There was a statistically significant difference in the frequency of febrile neutropenia episodes between patients receiving enteral nutritional support and those who did not receive such support (*p* = 0.049). However, this association should be interpreted cautiously and should not be considered causal, because enteral nutritional support may reflect underlying clinical severity, poor oral intake, mucositis, gastrointestinal toxicity, or treatment-related complications.

Total psoas muscle area at the L4–L5 level was evaluated in the 50 patients with suitable computed tomography images. Z-scores were calculated using the online calculator developed by Lurz et al. Based on these calculated z-score results, patients were classified as sarcopenic or non-sarcopenic. Forty of the 50 patients with available total psoas muscle area measurements were classified as sarcopenic, corresponding to 80% of the computed tomography-imaged subgroup. This percentage refers only to the imaged subgroup and should not be generalized to the entire cohort of 100 patients.

There was no statistically significant difference between patients with and without sarcopenia in terms of the number of febrile neutropenia episodes (*p* = 0.583), the frequency of severe neutropenia (*p* = 0.536), or febrile neutropenia-related mortality (*p* = 0.269). Similarly, no significant differences were observed between the groups regarding median age (*p* = 0.855), sex distribution (*p* = 0.560), body weight (*p* = 0.568), mean neutropenia duration (*p* = 0.568), or use of enteral nutritional support (*p* = 0.560).

In sarcopenia-specific analyses restricted to the 50 patients with available computed tomography-derived total psoas muscle area measurements, sarcopenia was not significantly associated with hospital stay. Therefore, patients without computed tomography-based sarcopenia assessment were not included as a separate comparison group for sarcopenia-related outcomes.

Exploratory multivariable analyses adjusted for age, sex, and body weight were performed. In exploratory Poisson regression analysis adjusted for age, sex, and body weight, enteral nutritional support was not independently associated with febrile neutropenia episode frequency in the overall cohort, although a borderline association was observed (IRR: 1.16, 95% CI: 1.00–1.36, *p* = 0.052). In the computed tomography-imaged subgroup, sarcopenia was not independently associated with febrile neutropenia episode frequency after adjustment for age, sex, and body weight (IRR: 1.20, 95% CI: 0.93–1.56, *p* = 0.168). In exploratory linear regression analysis, sarcopenia was not independently associated with hospital stay duration (β: 0.96 days, 95% CI: −2.85 to 4.76, *p* = 0.614). Because sarcopenia data were available only for 50 patients and the non-sarcopenic subgroup was small, these adjusted analyses were interpreted cautiously.

## Discussion

In pediatric cancer patients with a high risk of malnutrition, a comprehensive nutritional assessment should be conducted, and early nutritional support should be considered as part of supportive care. Nutritional education should be provided during hospitalization and at discharge, and individualized dietary interventions may be required according to the patient's clinical condition. Metabolic and nutritional interventions are important to support treatment tolerance and prevent deterioration in nutritional status (12). In low- and middle-income countries, undernutrition in children with cancer remains a major clinical problem, and its prevalence has been reported to be high in different pediatric oncology settings. Nutritional support has the potential to improve clinical outcomes, although its effect may vary depending on disease severity, treatment phase, and the presence of treatment-related complications (13).

The present study evaluated computed tomography-derived sarcopenia and enteral nutritional support in relation to febrile neutropenia episodes, neutropenia-related outcomes, hospital stay, and mortality in children treated for acute lymphoblastic leukemia. The main finding was that sarcopenia was common among patients with clinically indicated computed tomography images; however, sarcopenia was not significantly associated with febrile neutropenia episode frequency, severe neutropenia, febrile neutropenia-related mortality, or hospital stay in sarcopenia-specific analyses. Enteral nutritional support was associated with a higher febrile neutropenia burden in unadjusted analysis, but this association was not interpreted as causal.

In children with acute lymphoblastic leukemia, sarcopenia may develop during treatment as a result of systemic inflammation, prolonged hospitalization, reduced physical activity, corticosteroid exposure, and chemotherapy-related toxicity. This condition may increase vulnerability during follow-up and may contribute to reduced treatment tolerance (14). Chemotherapy can contribute to sarcopenia both directly and indirectly. Some chemotherapy agents may activate inflammatory pathways such as nuclear factor kappa-light-chain-enhancer of activated B cells, which regulates genes involved in inflammation, immune response, and cell survival. Activation of these pathways may contribute to muscle breakdown. Additionally, chemotherapy may increase oxidative stress and reactive oxygen species in skeletal muscle and may promote catabolic signaling through mediators such as transforming growth factor-beta (15). Indirectly, chemotherapy-related fatigue and reduced physical activity may further accelerate muscle loss.

Moreover, chemotherapy can damage rapidly dividing cells in the gastrointestinal tract (16). Children undergoing chemotherapy often experience diarrhea, nausea, vomiting, and mucositis, which can limit oral intake, cause fluid loss, and reduce nutrient absorption. Radiation therapy may contribute to muscle atrophy and long-term muscle and soft tissue loss, while corticosteroids may increase muscle protein breakdown and reduce new protein synthesis (17, 18). These mechanisms support the biological plausibility of muscle loss during pediatric leukemia treatment; however, the present study did not demonstrate an independent association between sarcopenia and febrile neutropenia burden. Therefore, our findings should be interpreted as exploratory and associative rather than causal.

In pediatric patients, the analysis of psoas muscle area from abdominal computed tomography imaging is becoming increasingly important in the evaluation of cancer-related sarcopenia. Total psoas muscle area may serve as an imaging-derived marker of low skeletal muscle mass. Lurz et al. established pediatric reference values for total psoas muscle area, defined as the sum of the right and left psoas muscle areas measured at the L3–L4 and L4–L5 intervertebral levels. They proposed age- and sex-specific percentile curves for total psoas muscle area at the L3–L4 and L4–L5 lumbar levels and developed an online calculator that generates z-scores for these measurements (11). Although total psoas muscle area was analyzed at both L3–L4 and L4–L5 levels in their study, the L4–L5 level has practical advantages in pediatric patients because the psoas muscle may have a rounder shape at this level, allowing more consistent contour delineation and improved interrater reliability. Moreover, the L4–L5 level is also commonly used for the assessment of visceral adipose tissue.

In the present study, sarcopenia was defined as a total psoas muscle area z-score below −2, based on age- and sex-specific reference values. Z-scores were calculated using the online calculator developed by Lurz et al., and patients were classified as sarcopenic or non-sarcopenic according to these calculated results. Among the 50 patients with available computed tomography-derived total psoas muscle area measurements, 40 patients were classified as sarcopenic, corresponding to 80% of the imaged subgroup. Importantly, this percentage refers only to patients with clinically indicated computed tomography images and should not be generalized to the entire cohort of 100 patients.

The high proportion of sarcopenia observed in the imaged subgroup may partly reflect selection bias. Computed tomography scans were performed for clinical indications and not for systematic research screening. Therefore, patients who underwent imaging may have represented a clinically more complicated or vulnerable subgroup. This issue limits the interpretation of sarcopenia prevalence and was one of the major methodological considerations in the present study.

Previous studies have suggested that low psoas muscle area may be associated with adverse outcomes in pediatric oncology. For example, children with low psoas muscle area z-scores have been reported to have poorer outcomes in some tumor groups (11, 19). Sarcopenia in pediatric patients may impair treatment tolerance, worsen functional reserve, and contribute to prolonged hospitalization (20). However, in our cohort, sarcopenia was not significantly associated with febrile neutropenia episode frequency, severe neutropenia, febrile neutropenia-related mortality, or hospital stay. This finding suggests that although computed tomography-derived sarcopenia may identify a vulnerable subgroup, its relationship with infectious complications in pediatric acute lymphoblastic leukemia requires further evaluation in larger prospective studies.

The interpretation of enteral nutritional support requires particular caution. In this study, enteral nutritional support was not used as a direct marker of malnutrition. Instead, it was analyzed as a supportive-care variable indicating the need for nutritional intervention during treatment. Patients receiving enteral nutritional support may have had more severe clinical problems, such as poor oral intake, mucositis, gastrointestinal toxicity, prolonged hospitalization, or more intensive treatment-related complications. Therefore, the observed unadjusted association between enteral nutritional support and higher febrile neutropenia episode frequency likely reflects confounding by indication rather than a harmful effect of enteral feeding itself. After exploratory adjustment for age, sex, and body weight, enteral nutritional support was not independently associated with febrile neutropenia episode frequency.

The febrile neutropenia-related mortality rate observed in this cohort was notable. However, because of the retrospective design, detailed data on treatment phase, microbiological documentation, infection source, intensive care requirement, and standardized cause-of-death adjudication were not uniformly available. Therefore, mortality findings should be interpreted cautiously. Future prospective studies should evaluate febrile neutropenia-related mortality using standardized definitions, microbiological data, and treatment-phase-specific analyses.

This study has several limitations. First, it was retrospective and conducted at a single center. Second, computed tomography-derived sarcopenia analysis was available only for 50 of 100 patients, creating a risk of selection bias. Third, formal nutritional assessment tools, such as body mass index-for-age z-scores, weight-for-age z-scores, mid-upper arm circumference, Subjective Global Nutritional Assessment, STRONGkids score, or serial weight-loss data during treatment, were not available for all patients. Therefore, this study cannot be considered a comprehensive evaluation of nutritional status. Fourth, enteral nutritional support may reflect underlying clinical severity rather than nutritional status itself. Fifth, although exploratory multivariable analyses adjusted for age, sex, and body weight were performed, the sample size and available variables were limited. Important potential confounders such as acute lymphoblastic leukemia risk group, chemotherapy protocol, cumulative corticosteroid exposure, mucositis severity, central venous catheter complications, microbiological findings, and socioeconomic factors were not uniformly available. Therefore, the findings should be interpreted as exploratory associations rather than causal relationships.

Despite these limitations, this study contributes to the growing literature on body composition assessment in pediatric acute lymphoblastic leukemia. The findings suggest that computed tomography-derived sarcopenia is common among imaged patients, but it was not independently associated with febrile neutropenia burden or hospital stay in the present cohort. Prospective studies using systematic imaging protocols, standardized nutritional assessments, formal malnutrition scores, treatment-related toxicity data, and adjusted statistical models are needed to clarify the clinical significance of sarcopenia and nutritional deterioration in children with acute lymphoblastic leukemia.

## Conclusion

In this retrospective cohort of children with acute lymphoblastic leukemia, computed tomography-derived sarcopenia was common among patients with clinically indicated imaging. However, sarcopenia was not independently associated with febrile neutropenia episode frequency or hospital stay after adjustment for age, sex, and body weight. Sarcopenia was also not significantly associated with severe neutropenia or febrile neutropenia-related mortality in unadjusted analyses.

Enteral nutritional support was associated with a higher number of febrile neutropenia episodes in unadjusted analysis; however, this relationship should be interpreted cautiously because enteral feeding likely reflects greater clinical severity and confounding by indication rather than a causal effect.

These findings highlight the need for systematic nutritional assessment and prospective studies incorporating standardized anthropometric measures, formal malnutrition scores, treatment-related toxicity data, and adjusted analyses in children with acute lymphoblastic leukemia. Future studies are needed to clarify the clinical significance of sarcopenia and nutritional deterioration in this vulnerable population.

## Data Availability

The raw data supporting the conclusions of this article will be made available by the authors, without undue reservation.
